# Editorial: Systemic inflammation in severe infectious diseases

**DOI:** 10.3389/fimmu.2024.1483682

**Published:** 2024-09-09

**Authors:** Michal Holub, Julien Pottecher, Heiko Herwald, Mukesh Pasupuleti, Praveen Papareddy

**Affiliations:** ^1^ Department of Infectious Diseases, First Faculty of Medicine, Charles University and Military University Hospital Prague, Prague, Czechia; ^2^ Hôpitaux Universitaires de Strasbourg, Service d’Anesthésie-Réanimation & Médecine Péri-opératoire - Université de Strasbourg, FMTS, UR 3072, Strasbourg, France; ^3^ Division of Infection Medicine, Department of Clinical Sciences, Lund University, Lund, Sweden; ^4^ Division of Molecular Microbiology & Immunology, CSIR-Central Drug Research Institute, Lucknow, Uttar Pradesh, India; ^5^ AcSIR: Academy of Scientific and Innovative Research (AcSIR), Ghaziabad, India

**Keywords:** inflammation, immunity, sepsis, biomarker, COVID

## Introduction

Systemic inflammation is a double-edged sword in the context of severe infectious diseases. While it is a critical component of the body’s defense mechanism, its dysregulation can lead to severe complications, including multi-organ failure and death. The research compiled in this Research Topic “Systemic Inflammation in Severe Infectious Diseases” provides an in-depth examination of the mechanisms, biomarkers, and therapeutic strategies aimed at understanding and mitigating the detrimental effects of systemic inflammation. This editorial aims to weave these contributions into a cohesive narrative, highlighting their interconnectedness and overall impact on the field.

## The underlying mechanisms of systemic inflammation

At the heart of systemic inflammation is the intricate dance between the host’s immune response and the invading pathogens. This complex interplay is vividly illustrated in the work of Calvier et al., who explored the role of circulating Reelin in COVID-19. Their study revealed that elevated levels of Reelin correlate with increased disease severity, suggesting that Reelin not only serves as a biomarker but also a potential therapeutic target. This finding underscores the broader theme that biomarkers of inflammation are not merely indicators of disease but active participants in the pathogenic process.

Building on this theme, Wang and He delve into the role of Gasdermins in sepsis. Gasdermins, as pore-forming proteins, are pivotal in the release of pro-inflammatory mediators and the execution of inflammatory cell death. Their review highlights the therapeutic potential of targeting Gasdermins to control the systemic inflammatory response, thereby preventing multi-organ dysfunction—a common thread in the pathogenesis of severe infections like sepsis and COVID-19.

## Biomarkers as predictive tools and therapeutic targets

A recurrent theme in this Research Topic is the identification and validation of biomarkers that can predict disease severity and guide therapeutic interventions. The work by Mangoni and Zinellu on the systemic inflammation index (SII) exemplifies this approach. Their meta-analysis demonstrated that higher SII values are significantly associated with severe COVID-19 and mortality, positioning SII as a crucial tool for early risk stratification. This concept is further enriched by the study of Zhang et al., who identified key RNA methylation-associated genes (RMGs) that classify sepsis into distinct subtypes, offering new avenues for diagnosis and treatment.

The exploration of biomarkers extends beyond their predictive value. Bourcier et al. investigated the role of beta1-adrenergic blockers in systemic inflammation, showing that these blockers can inhibit extracellular trap formation and preserve neuromuscular function. Their findings suggest that targeting biomarkers and their associated pathways can not only predict disease outcomes but also serve as therapeutic interventions.

In the context of distinguishing sepsis from trauma-induced sterile inflammation, Papareddy et al. identified a panel of biomarkers that can effectively differentiate between these conditions. Their study uncovered distinct protein patterns associated with early trauma-induced sterile inflammation and sepsis, with SYT13 and IL1F10 emerging as potential early sepsis biomarkers. This research highlights the importance of accurate differentiation in clinical settings to ensure appropriate treatment strategies.

## Therapeutic strategies to mitigate inflammation

Moving from biomarkers to therapeutic strategies, the research highlights several innovative approaches to modulate the inflammatory response. Zemtsovski et al. demonstrated that alpha1-antitrypsin (AAT) treatment improves survival in a murine model of abdominal sepsis by reducing inflammation and sequestering free heme. Their findings not only highlight the protective effects of AAT but also suggest its potential as a therapeutic agent in sepsis, where uncontrolled inflammation is a major driver of morbidity and mortality.

Another promising therapeutic strategy is illustrated by Ghimire et al., who investigated the role of NLRP6 in polymicrobial sepsis. They found that NLRP6 knockout mice exhibited enhanced survival and reduced bacterial burden, suggesting that NLRP6 may act as a negative regulator of host defense. This discovery opens up new possibilities for developing therapies that target specific components of the inflammatory response to improve outcomes in sepsis patients.

## Future directions and emerging concepts

The quest to understand and manage systemic inflammation in severe infectious diseases is ongoing, with new concepts continuously emerging. Song et al. provide a comprehensive review of the immune reconstitution inflammatory syndrome (IRIS) in Whipple disease, emphasizing the exaggerated inflammatory responses that occur during immune reconstitution. Their insights into the pathogenesis of IRIS and potential monitoring tools highlight the complexity of managing systemic inflammation in the context of immune reconstitution.

Moreover, the study by Wu et al. on the neutrophil-to-lymphocyte ratio (NLR) reinforces the utility of this biomarker in predicting prognosis and mortality in sepsis patients. Their meta-analysis supports the notion that simple, readily available biomarkers can have profound implications for clinical decision-making and patient outcomes.

The emerging role of lipid metabolism in systemic inflammation is another fascinating area explored in this Research Topic. Muniz-Santos et al. discuss how lipid oxidation dysregulation contributes to the pathophysiology of sepsis. They suggest that fatty acid mobilization and oxidation changes can serve as valuable markers for sepsis diagnosis and prognosis, providing new insights into the metabolic aspects of inflammatory responses.

## Conclusion

The articles in this Research Topic collectively advance our understanding of systemic inflammation in severe infectious diseases. They highlight significant mechanisms, identify crucial biomarkers, and explore innovative therapeutic strategies. This cohesive narrative underscores the interconnectedness of these studies and their collective impact on improving the diagnosis, management, and treatment of systemic inflammation in severe infectious diseases. The ongoing research and emerging concepts presented here lay a robust foundation for future studies aimed at mitigating the adverse effects of systemic inflammation and enhancing patient outcomes in this challenging field.

By weaving together the threads of mechanistic insights, biomarker discovery, and therapeutic innovation, this Research Topic provides a comprehensive and forward-looking perspective on systemic inflammation in severe infectious diseases. We hope these contributions will spur further research and clinical applications, ultimately leading to better strategies for managing and treating these complex conditions.

